# Integration of large-scale data for extraction of integrated *Arabidopsis* root cell-type specific models

**DOI:** 10.1038/s41598-018-26232-8

**Published:** 2018-05-21

**Authors:** Michael Scheunemann, Siobhan M. Brady, Zoran Nikoloski

**Affiliations:** 1 Systems Biology and Mathematical Modeling Group, Max Planck Institute of Molecular Plant Physiology, Potsdam-Golm, Germany; 2 Department of Plant Biology and Genome Center, University of California, Davis, CA 95616 USA; 3 Bioinformatics Group, Institute of Biochemistry and Biology, University of Potsdam, Potsdam-Golm, Germany

## Abstract

Plant organs consist of multiple cell types that do not operate in isolation, but communicate with each other to maintain proper functions. Here, we extract models specific to three developmental stages of eight root cell types or tissue layers in *Arabidopsis thaliana* based on a state-of-the-art constraint-based modeling approach with all publicly available transcriptomics and metabolomics data from this system to date. We integrate these models into a multi-cell root model which we investigate with respect to network structure, distribution of fluxes, and concordance to transcriptomics and proteomics data. From a methodological point, we show that the coupling of tissue-specific models in a multi-tissue model yields a higher specificity of the interconnected models with respect to network structure and flux distributions. We use the extracted models to predict and investigate the flux of the growth hormone indole-3-actetate and its antagonist, trans-Zeatin, through the root. While some of predictions are in line with experimental evidence, constraints other than those coming from the metabolic level may be necessary to replicate the flow of indole-3-actetate from other simulation studies. Therefore, our work provides the means for data-driven multi-tissue metabolic model extraction of other *Arabidopsis* organs in the constraint-based modeling framework.

## Introduction

Plant organs are not homogeneous, but are composed of multiple cell types which are organized in space and time to facilitate adequate functions^1^. For instance, the root of *Arabidopsis thaliana* is composed of 15 cell types^2^. The cell types across developmental stages also differ with respect to their metabolic and regulatory characteristics, and they, too, are organized geometrically with respect to specific radial and axial positions. For instance, different developmental zones (e.g., meristematic, elongation and maturation zone) can be separated along the longitudinal axis of the root, with more matured cells in the zone farther away from the tip^3^. Therefore, there is a need for models of connected tissues to investigate metabolism on the level of an organ and entire organism^4^. Such an approach will allow us to probe how root cell types complement and limit each other in fulfilling their respective functions, and will provide insights about how the root sustains the functions of the entire plant.

Genome-scale models (GEMs), comprising the entirety of characterized metabolic reactions, together with constraint-based modeling methods offer the means to address this question^5^. The availability of GEMs of the well-studied model plants (e.g., for *Arabidopsis thaliana*^6–9^) and the public accessibility of spatiotemporally-resolved “omics” data (e.g., for *Arabidopsis thaliana* roots^3,10^) render it feasible to develop tissue-specific models for plant root system. To this end, given a plant GEM, context-specific models can be obtained by extracting a subset of reactions relevant for a particular context, i.e., cell type, developmental stage or a tissue.

A recent systematic review^11^ grouped existing context-specific model construction approaches into three categories, referred to as GIMME-, iMAT- and MBA-like families, which now includes a forth category for the recent RegrEx^12^ method as well as for the approach provided by Lee *et al*.^13^ named Lee2012. Representative methods from the first three categories have been thoroughly reviewed and their performance with respect to prediction of growth and particular fluxes compared ion several data scenarios^14^. The underlying concept of GIMME-like methods (e.g., GIMME^15^, GIM^3^E^16^) is a two-step procedure: a requiring metabolic functionality (RMF), such as growth, is first optimized in a flux balance analysis (FBA) framework^17^. Subsequently, a penalty function is minimized, such that the discrepancy between fluxes and respective transcripts level is small at the optimal biomass (or fraction thereof) detected at step one. The differences between methods in this category stems from the formulation of the discrepancy measure. The iMat-like family (e.g., iMAT^18^, INIT^19^) is characterized by finding reactions whose states (active/ inactive) correspond to the respective states from the data, i.e. expressed/ non-expressed gene(s) encoding enzymes that catalyze the considered reaction. It is mathematically formulated as mixed integer linear program (MILP) which aims to find a steady-state flux distribution, while the number of reactions whose activity states meet the expression states is maximized. For the third construction strategy (e.g., MBA^20^, mCADRE^21^), the reactions of generic model are *a priori* divided into two groups, namely core and non-core reactions with respect to existing evidence (i.e., high-throughput data or biochemical knowledge). Next, a pruning process takes place in which non-core reactions are removed that are not needed for enabling the core reactions to be active (i.e., consistency achieved by gap filling). Based on the associated transcriptomics or proteomics data, the partition of the reactions into groups is usually based on user-chosen thresholds^11^.

Identifying an appropriate threshold can be difficult in non-model organisms, especially when information about context-specific metabolic functions is lacking, since usage of mean or median summary statistics may bias the results and may impose challenges in the comparability of contexts. The Regularized Context-specific model Extraction method (RegrEx) provides a possibility for fully automated extraction of tissue-specific models by finding a compromise between sparsity of flux distributions (i.e., number of active reactions) and flux distribution that minimizes the distance to the transcriptomics data^12^ (see Materials and Methods for details about RegrEx and its comparison to other existing approaches for context-specific metabolic modeling). In contrast to the existing approaches based on known functions, RegrEx does not optimize any pre-selected biological functionality, which may render it suitable for analysis of poorly understood metabolic scenarios. The lack of functional consideration may be partly overcome by consideration of qualitative metabolite data (i.e. presence/absence patters) in a cell or tissue to further refine tissue-specific models. To this end, one can use approaches such as GIM^3^E^16^, whereby measured metabolites are incorporated in the model by enforcing a non-zero flux through specially designed sink reactions. In addition, one may use minExCard^22^, which minimizes the number of added exchange reactions that renders a feasible steady-state flux around measured metabolites. Here, we expand RegrEx^12^ to include qualitative metabolite data, i.e., present/absent, following the idea of GIM^3^E^16^.

To obtain insights in the organization of metabolism at a larger scale, tissue-specific models can be combined in a multi-tissue model. In plant science, there already exist several multi-tissue modeling approaches: De Oliveira Dal’Molin *et al*. provide a framework to build multi-tissue models for an entire plant^23^. This framework relies on additional compartments connecting two adjacent tissues; the compartments comprise common pools through which exchanged metabolites must move. Simulation of steady-state fluxes uses the assumption that organs do not compete for energy demand which is supposed to be minimized on the level of a whole plant. Grafahrend-Belau *et al*.^24^ proposed a slightly different strategy: In this approach, the authors focused on modeling the primary metabolism of leaf, stem and seed. These tissue-specific models were then connected by an additional compartment, the phloem, which allows communication between tissues without taking into account any geometrical cellular organization. The tissue-specific models were created by utilizing genomic, proteomics, biochemical, and physiological data from literature and publicly accessible databases, without employing the approaches for tissue-specific network construction mentioned above. The approaches followed in both studies require prior biological knowledge about the function of individual organs or their underlying principles, which are often unknown. In absence of knowledge of optimized metabolic function, approaches based on integrating diverse data sets (e.g. transcriptomics, proteomics, and metabolomics) to approximate functional (i.e. flux) states may provide a viable alternative.

**Figure 1:**
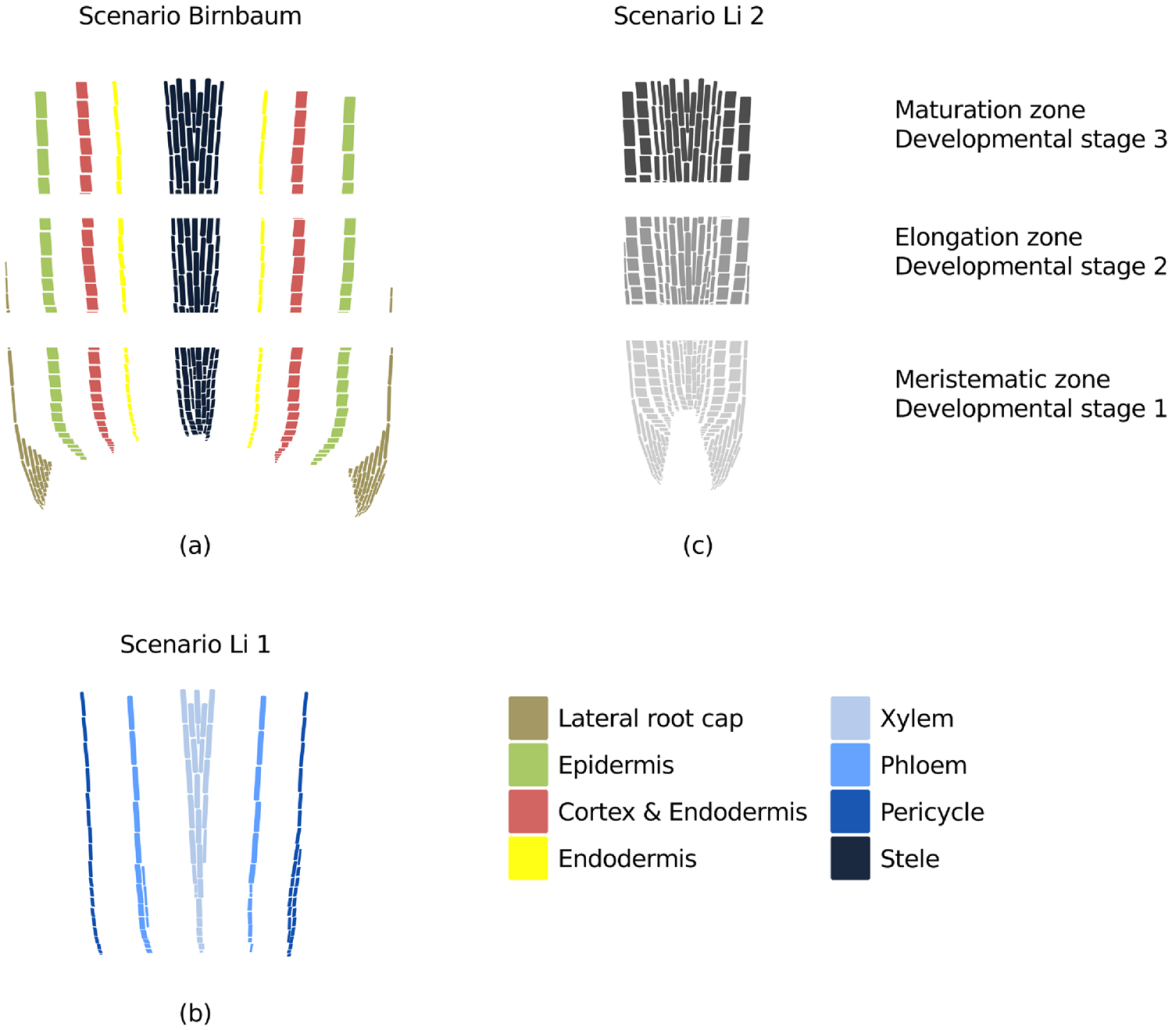
Data scenarios for the model extraction approach. (**a**) In the Birnbaum scenario, spatio-temporally resolved transcriptomics data were used for the RegrEx method to extract (de)coupled tissue-specific models. The data sets were obtained from Birnbam *et al*.^3^. The considered cell types or tissue included lateral root cap, epidermis, cortex & endodermis as well as endodermis cells and cells composing the stele tissue (from outer to inner layers) of three developmental stages, i.e. meristematic, elongated and matured cells. The multi-tissue model was formed along the longitudinal axis of each cell type/tissue. (**b**) The Li 1 scenario comprised models covering the xylem, phloem and pericycle cells that were forming a stele multi-tissue model. (**c**) In the Li 2 scenario, only the developmental stages were considered. The multi-tissue model was simulating the root organ. For (b) and (c) transcriptomics data were obtained from Li *et al*.^25^.

The aim of this study was to form root models that combine cell type or tissue-specific networks for three scenarios corresponding to three experimental set ups: (i) the Birnbaum scenario was based on the data assembled in Birnbaum *et al.*^3^. which mapped gene expression for 15 different zones of *Arabidopsis* root, corresponding to the stele tissue as well as for endodermis, cortex & epidermis (atrichoblasts) cells at three progressive developmental stages (Fig. 1a). (ii) the Li 1 scenario was based on the data from Li *et al*.^25^ which provides gene expression for (cell type-resolved) xylem, phloem and pericycle cells (Fig. 1b), while (iii) the Li 2 scenario was based on gene expression data of Li *et al*.^25^ obtained from the mixture of cells in the meristematic, elongation and maturation zone of the root (Fig. 1c), allowing us to extract developmental-stage-resolved models. Meristematic, elongation and maturation zones are equivalent to developmental stages 1 to 3, respectively. We will abuse the language, and will refer to the respective models as tissue-specific models. Our strategy for extracting tissue-specific as well as multi-tissue models was based on RegrEx^12^ (using transcriptomics data) with the addition of considering exchange reactions connecting the tissue-specific models. In addition, we extended RegrEx to allow the integration of qualitative metabolomics data from Moussaieff *et al*.^26^ Therefore, the Birnbaum scenario provides the biggest cell and development resolution, while the Li 1 and 2 scenarios can be used to verify the robustness of the predicted patterns.

The resulting (de)coupled models were compared with each other in terms of their structure as well as the biological functionality, particularly the correspondence of the supported flux distributions as well as the proxy of turnover for key hormones. Given two model extraction strategies applied to extract a set of models, one will be said to extract more specific models if the pairwise difference of the models with respect to different measures is larger. As measures for model differences one can use the difference in model structure (i.e., in reactions or metabolites) or in model functionality (i.e., in flux distributions). With the data at hand, our results suggested that extraction of coupled models resulted in a higher specificity of the extracted tissue-specific models than the decoupled models. In addition, we considered qualitative proteomics data from Petricka *et al*.^27^ to qualitatively verify some of the predictions. Our results also indicated that the presented approach may be suitable to investigate phytohormone distributions such as indole-3-actetate (IAA) and trans-zeatin as antagonist to IAA^28^ in the root. Therefore, our modeling strategy could be used to extract networks of tissues with higher specificity for other plant organs as more spatiotemporal data become available.

## Results and Discussion

**Figure 2:**
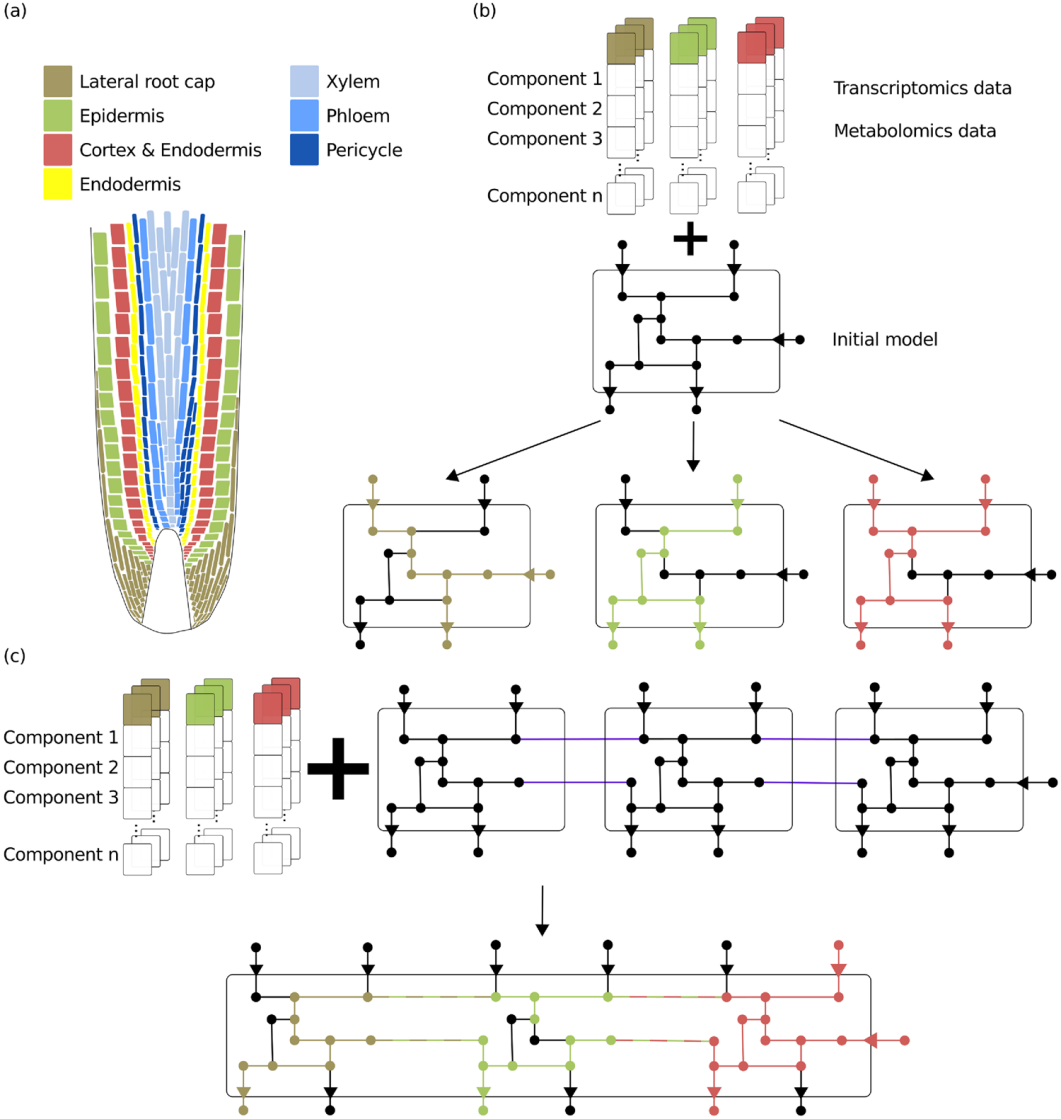
Cell-type specific model extraction for Arabidopsis root. (**a**) Types of tissues (stele) and cell types, i.e. epidermis, cortex, endodermis and lateral root cap. (**b**) For tissue/cell type-specific transcriptomics data, decoupled models for every single tissue are extracted from an initial generic model. Vertices represent metabolites, lines stand for reactions. Transcriptomics and metabolomics data for the components, i.e., genes and metabolites, are used for model extraction. Colored lines correspond to reactions that are extracted from the initial model. The colors correspond to the respective tissue. (**c**) The replicates of the initial model are connected to each other via exchange reactions. A multi-tissue model is then extracted based on the corresponding transcriptomics data. The purple colored lines show the reactions connecting the multi-tissue model. The single-tissue specific parts within the multi-tissue model refer the coupled models, and are represented in different colors. Note that the decoupled and coupled models may differ.

The RegrEx^12^ approach was applied to extract models specific to cell types or tissues depicted in Fig. 1, from an initial generic GEM based on transcriptomics and metabolomics data in *Arabidopsis* root (Fig. 2a). A context-specific model extracted only by using transcriptomics data from the respective cell type will be referred to as *decoupled* (Fig. 2b). In contrast, a tissue-specific model extracted by integration of transcriptomics data from multiple cell types in a multi-context model will be called *coupled* (Fig. 2c). To evaluate the performance of RegrEx for the two types of extracted context-specific models (i.e., decoupled and coupled) as well as the multi-context model, we employed the Pearson correlation coefficients between the data (i.e., gene expression and protein abundance) and the flux distributions resulting from RegrEx as well as sampled from the extracted models. For fairness of the comparison between the coupled and decoupled tissue-specific models, here, exchange reactions (i.e., reactions through which metabolites are transported between two models) were not considered since these reactions are, expectedly, over-represented in the coupled tissues.

### Properties of decoupled and coupled context-specific models

**Figure 3:**
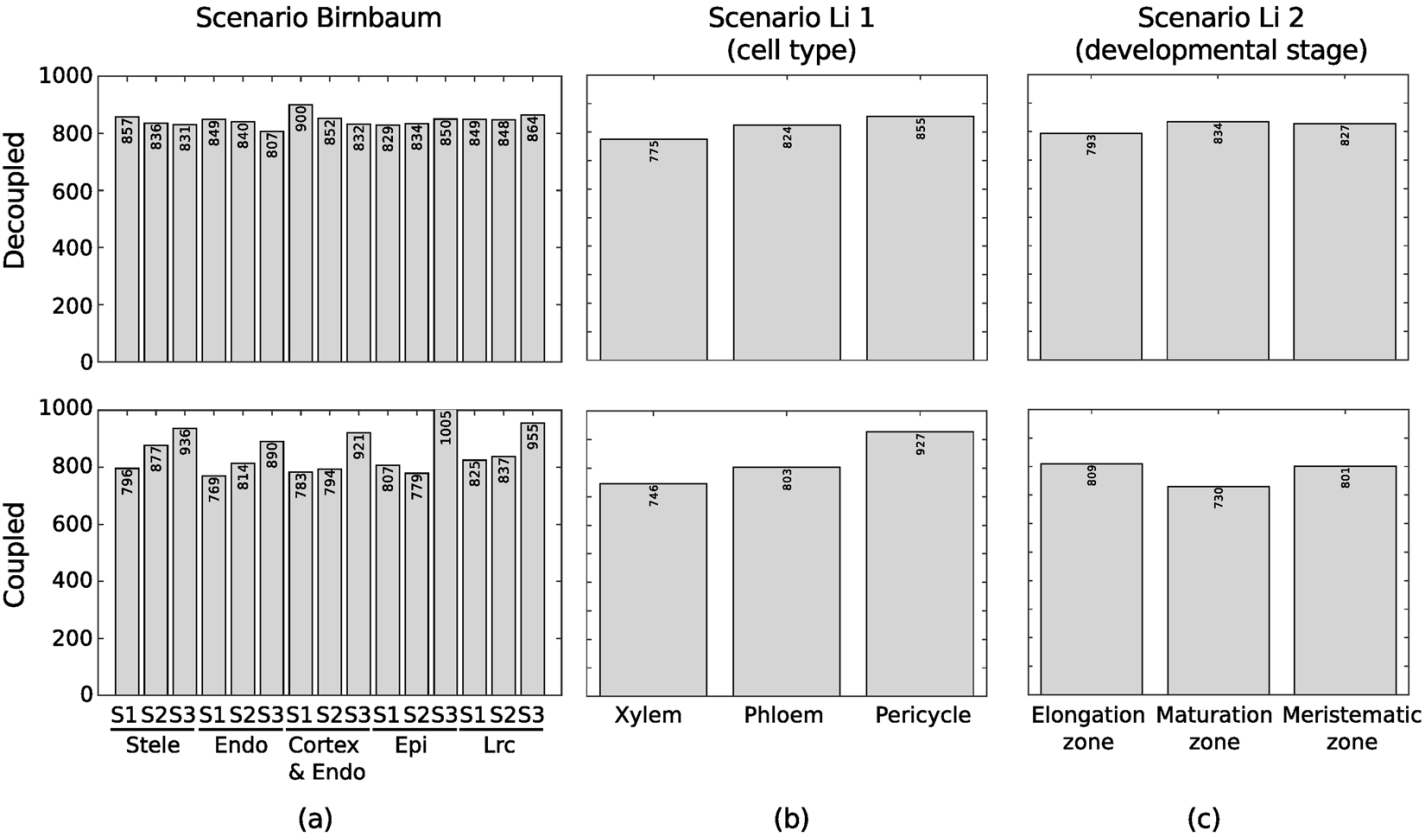
Number of extracted reactions. The number of reactions that are extracted from the initial model are given for three situations: (**a**) Transcriptomics data published by Birnbaum *et al*. are considered. The data is spatiotemporal resolved and covers the stele tissue and the cell-types endodermis (endo), cortex & endodermis (cortex/endo), epidermis (epi) and lateral root cap (lrc) for three developmental stages 1 (S1), the region between the root tip and 0.15 mm upwards (where the full diameter of the primary root is reached, stage 2 (S2), where cells originating from the root section between 0.15 mm to 0.30−0.45 mm away from the root tip, and stage 3 (S3), that is about 0.45 mm to 2 mm far away from the root tip. (**b**) Transcriptomics data published by Li *et al*.^25^ are used spatial (cell-type) and (**c**) temporal (developmental stage) resolution.

To obtain insights into the structure of the resulting decoupled models, we compared the sets of extracted reactions. Each of the decoupled context-specific models contained up to 45% (Fig. 3) of the reactions from the initial GEM (with 2,199 reactions). The models extracted by both strategies were of comparable compactness, assessed by the number of considered reactions (Fig. 3). The largest difference between the number of extracted reactions between the decoupled and coupled models in Scenario Birnbaum was for matured epidermis cells, while for Scenarios Li 1 and 2, these included the pericycle cells and matured root cells, respectively.

Next, we were also interested in how well the predicted fluxes matched the transcriptomics data used for the model extraction. To this end, the Pearson correlation between the transcriptomics data and the predicted flux distributions were determined. We would like to note that the RegrEx approach minimizes the distance between gene expression and flux values which does not correspond to maximizing correlation. It can be observed, in Table 1 that the correlation values between fluxes and transcriptomics data were slightly lower for the coupled models compared to the decoupled in the scenarios Li 1 and Li 2, except for the pericycle cell type. In the Birnbaum scenario, the Pearson correlation coefficients for the coupled tissue-specific models were at least as large as the correlation values for the decoupled models. For instance, the correlation coefficients between fluxes and trancriptomics data were equal for (de)coupled models describing cortex & endodermis tissue of the maturation zone (developmental stage 3). For the meristematic zone of the same tissue, the correlation was increased by 37% when coupling was considered, from 0.19 to 0.26. In contrast, the model representing endodermis cells behaved differently. For the meristematic zone, the Pearson correlation decreased by 21% when coupling was considered. The Pearson correlation coefficient also decreased for lateral root cap cells of meristematic zone from 0.38 to 0.37. Except for these three particular cases, the Pearson correlation increased on average by 8% when coupling was considered. Altogether, we concluded that the Pearson correlation for the coupled tissue-specific models was comparable to the decoupled tissue-specific models on the three analyzed scenarios (Table 1).

**Table 1:** Concordance of flux distributions and the transcriptomics data.

		decoupled	coupled
elongation zone		0.40	0.34
maturation zone		0.39	0.31
meristematic zone		0.19	0.15
xylem		0.39	0.37
phloem		0.40	0.37
pericycle		0.37	0.38
meristematic zone		0.29	0.30
elongation zone	stele	0.37	0.37
maturation zone		0.47	0.49
meristematic zone		0.20	0.20
elongation zone	endodermis	0.41	0.42
maturation zone		0.44	0.45
meristematic zone		0.19	0.26
elongation zone	cortex & endodermis	0.35	0.36
maturation zone		0.40	0.40
meristematic zone		0.19	0.15
elongation zone	epidermis	0.34	0.35
maturation zone		0.40	0.40
meristematic zone		0.38	0.38
elongation zone	lat root cap	0.39	0.42
maturation zone		0.50	0.53

For all three scenario Birnbaum, Li 1 and Li 2 the flux distributions are compared with the respective transcriptomics data by determining the Pearson correlation coefficient. All presented correlations were significant at level 0.001.

### Differences in network structure and flux distributions highlight a higher specificity of coupled models

**Figure 4:**
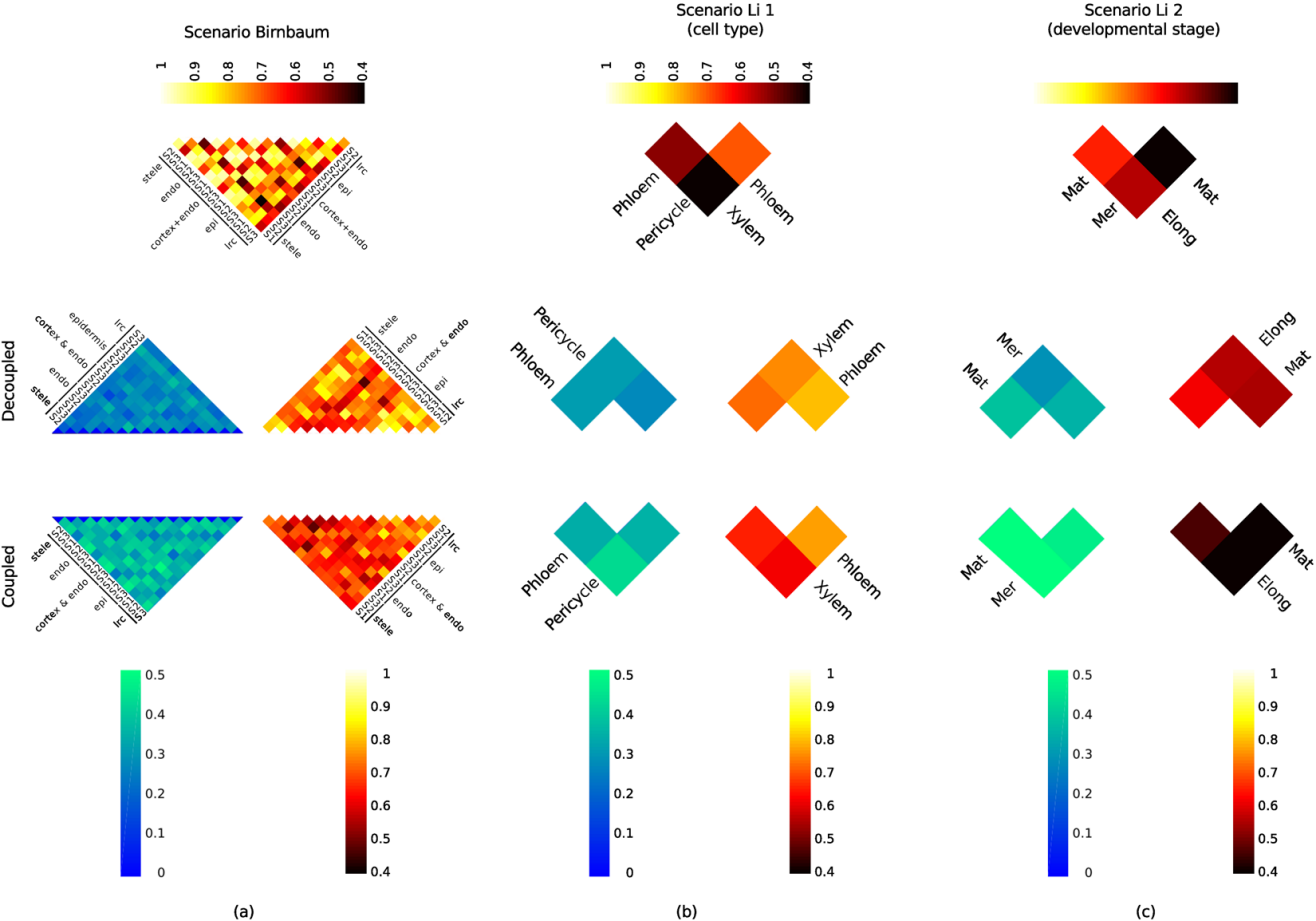
Structural and functional comparison of extracted models. For three scenarios the decoupled models were compared with coupled models: (**a**) scenario Birnbaum (only transcriptomics data from Birnbaum *et al*. are used) (**b**) scenario Li 1 in which transcriptomics data for different cell types from Li *et al*. were considered and (**c**) scenario Li 2 in which the gene expression data for three developmental stages were considered. The heat maps on the left side correspond to the Jaccard distances, while those on the right side refer to the Pearson correlation of the flux distributions.

First, we inspected the concordance between the developmentally-resolved transcriptomics data. To this end, we determined the pairwise Pearson correlation based on the gene expression for each pair of tissues by considering only the genes included in the initial, generic GEM. From the uppermost triangle-shaped heat map of Fig. 4a and c, it was apparent that the transcriptomics data of the more matured tissue (i.e., for scenario Birnbaum, developmental stage 3 referred to as DS3, and scenario Li, developmental stage resolved: matured denoted by Mat) differed the most from the cells or tissue of the earlier developmental stages. This was in line with the biological role of more matured cells/tissues that perform more specific tasks^29,30^.

Next, we inspected if and to what extent this relationship was mirrored when considering the cohorts of the decoupled and of the coupled models, respectively. The structure of the models was compared by employing the Jaccard distance. The Jaccard distance ranges between 0 and 1, where 1 indicates that two pairwise compared models have no reaction in common. From the heat maps on the left-side of Fig. 4 (blue-scaled), it was noticeable that contexts that were more matured showed higher Jaccard distances compared to the less matured contexts for the (de)coupled models. More precisely, the pairwise Jaccard distance increased along root development (longitudinal axis). However, the models for the meristematic tissues differed slightly from those of the matured tissue.

Subsequently, we performed a comparison of the models by considering the pairwise Pearson correlation between the predicted flux distributions. We will refer to such comparison as functional, as it is based on the supported flux distributions. The Pearson correlations obtained values between −1 and 1, where 1 reveals that two considered flux distributions fit each other perfectly. From the heat maps on the right side of Fig. 4, when considering the decoupled models, we found that all flux distributions were more concordant to each other in the Birnbaum and Li 2 scenarios. In contrast, the coupled models exhibited smaller Pearson correlation coefficient. For instance, for the decoupled epidermis-specific models the Pearson correlation coefficients were 0.89, 0.83 and 0.85 for developmental stages 1 and 2, 1 and 3, and 2 and 3, respectively. For the coupled models, the values of the Pearson correlation were 0.75, 0.66, and 0.79 for the three comparisons for developmental stages, respectively. This pattern was stable across all considered tissues/cell types, indicating that the coupled models are more specific. In other words, the extracted flux distributions were less concordant across all models, indicating that the models were functionally more different than their decoupled counterparts.

**Figure 5:**
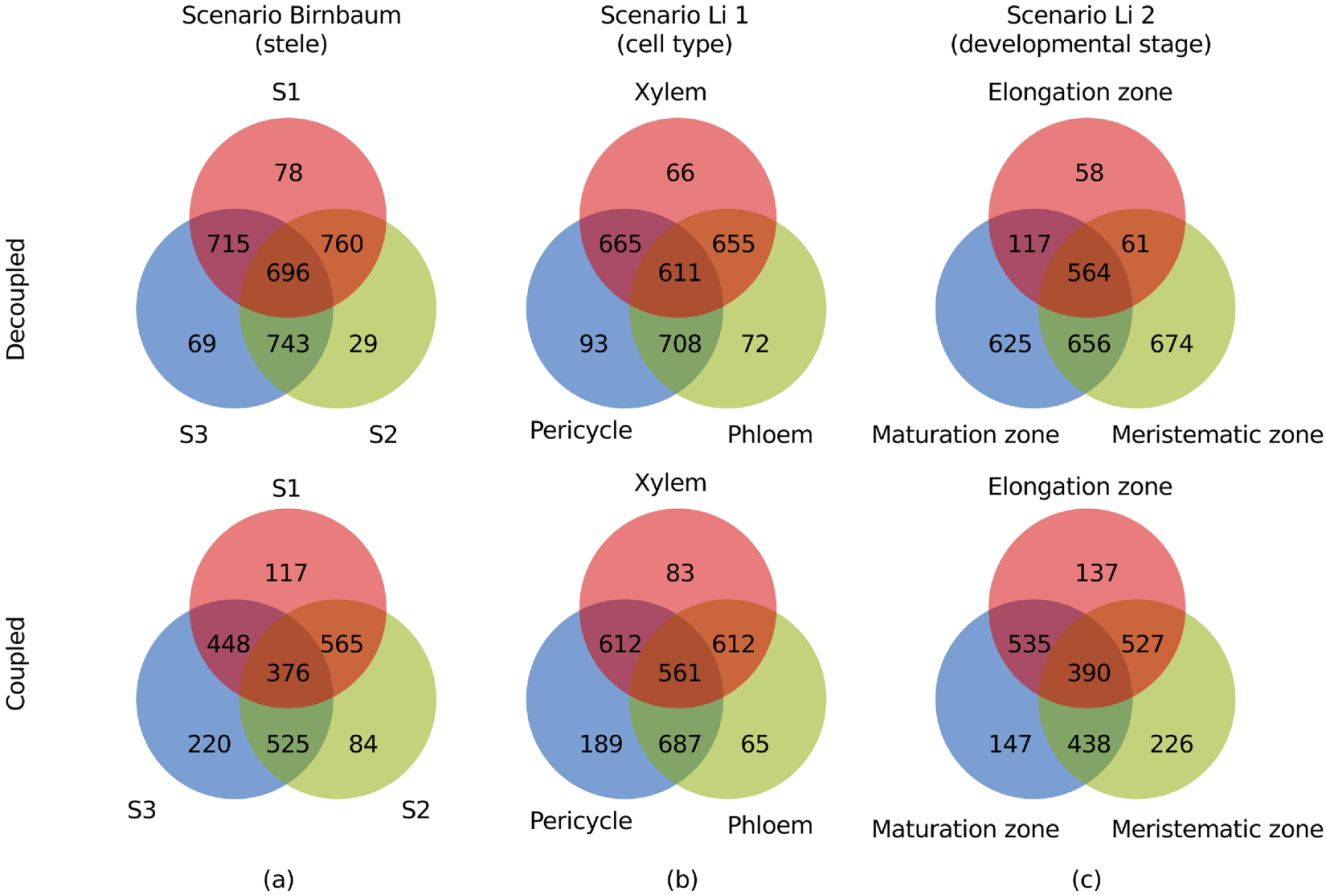
Context-specific reactions and reactions shared across contexts. Venn diagram of pairwise shared, individual reactions and reactions that are shared by all for three scenarios (**a**) scenario Birnbaum (only transcriptomics data from Birnbaum *et al*. are used) and the stele context only. S1, S2, S3 refer to developmental stage 1, 2 and 3, respectively, that is equivalent to meristematic, elongation and maturation zone, (**b**) scenario Li 1 in which transcriptomics data for different cell types from Li *et al*. were considered and (**c**) scenario Li 2 in which the gene expression data for three developmental stages were considered.

We were also interested in investigating how many reactions were shared pairwise and among all extracted (de)coupled models per context. As shown in Fig. 5, we found that for the coupled models the number of reactions shared by all contexts decreased in all three scenarios (in Scenario Birnbaum, Li 1 and 2, by 15%, 8%, and 31%, respectively). In contrast, the number of context-specific reactions increased, except for the phloem cell type in scenario Li 1 and for stele cells of developmental stage 1 in the Birnbaum scenario. The same trend was observed in scenario Li 2. These findings provided further support for the claim that the coupled context-specific models resulted in a higher specificity compared to the decoupled models.

### Sampling-based comparison of decoupled and coupled tissue-specific models

We also wanted to compare the specificity of the models by comparing the randomly sampled flux distributions for decoupled and coupled tissue-specific models over the shared reactions. This comparison reflects the functional similarities of the models. To this end, flux values were uniformly sampled for the extracted (de-)coupled context-specific models using the implementation by Schellenberger *et al*.^31^ (see Methods). The pairwise Pearson correlation was then determined for 2,500 sampled flux distributions from each of the models. The resulting correlation matrices were compared with each other by employing the *R*_*v*_-coefficient^32^ (Figure S1). Analogous to the Pearson correlation coefficient, the *R*_*v*_-coefficient is a measure of similarity of matrices and takes values between 0 and 1. Tables 2–4 showed that for the coupled tissue-specific models the R_v_-coefficient was considerably smaller compared with the decoupled models for all three scenarios (Table 2: Li 1, Table 3: Li 2 and Table 4: Birnbaum). The decreasing similarity of pairwise considered correlation matrices demonstrated again that the coupled context-specific models were more specific than their decoupled counterparts.

**Table 2:** Comparison of the predicted and the sampled flux distributions for the Li 1 scenario.

	xylem	phloem	pericycle
xylem		**0**.**84**	**0**.**83**
phloem	*0*.*48*		**0**.**93**
pericycle	*0*.*53*	*0*.*59*	

For the extracted models flux values were randomly sampled for each model. The comparison of the models is conducted only based on the reactions shared between the models in each of the groups of coupled and decoupled to facilitate unbiased comparison of specificity. The comparison is based on the R_v_-coefficient which ranges between 0 and 1. Similar to the Pearson correlation an R_v_-coefficient of 1 means high similarity. Bold font indicates values corresponding to the decoupled models, whereas the italic font corresponds to the coupled models.

**Table 3:** Comparison of the predicted and the sampled flux distributions for the Li 2 scenario.

	meristematic	elongated	maturated
meristematic		**0**.**84**	**0**.**79**
elongated	*0*.*53*		**0**.**79**
maturated	*0*.*51*	*0*.*79*	

For the extracted models flux values were randomly sampled for each model. The comparison of the models is conducted only based on the reactions shared between the models in each of the groups of coupled and decoupled to facilitate unbiased comparison of specificity. The comparison is based on the R_v_-coefficient which ranges between 0 and 1. Similar to the Pearson correlation an R_v_-coefficient of 1 means high similarity. Bold font indicates values corresponding to the decoupled models, whereas the italic font corresponds to the coupled models.

**Table 4:** Comparison of the predicted and the sampled flux distributions for the Birnbaum scenario.

	meristematic	elongated	maturated	
meristematic		**0**.**82**	**0**.**80**	
elongated	*0*.*61*		**0**.**91**	stele
maturated	*0*.*61*	*0*.*68*		
meristematic		**0**.**74**	**0**.**71**	
elongated	*0*.*57*		**0**.**75**	endodermis
maturated	*0*.*43*	*0*.*56*		
meristematic		**0**.**82**	**0**.**73**	
elongated	*0*.*57*		**0**.**75**	cortex & endodermis
maturated	*0*.*43*	*0*.*56*		
meristematic		**0**.**71**	**0**.**63**	
elongated	*0*.*26*		**0**.**86**	epidermis
maturated	*0*.*31*	*0*.*58*		
meristematic		**0**.**74**	**0**.**61**	
elongated	*0*.*29*		**0**.**75**	lat root cap
maturated	*0*.*11*	*0*.*29*		

For the extracted models flux values were randomly sampled for each model. The comparison of the models is conducted only based on the reactions shared between the models in each of the groups of coupled and decoupled to facilitate unbiased comparison of specificity. The comparison is based on the R_v_-coefficient which ranges between 0 and 1. Similar to the Pearson correlation an R_v_-coefficient of 1 means high similarity. Bold font indicates values corresponding to the decoupled models, whereas the italic font corresponds to the coupled models.

### Validation with proteomics data

We also evaluated the resulting tissue-specific models by using data from another cellular layer, namely, proteomics data^25,27^. Firstly, we compared the proteomics data with the transcriptomics data using the Pearson as well as the Spearman correlation coefficients. We also compared the structure of the models extracted based loci for which both transcriptomics and proteomics data were available. In total, proteomics data could be mapped to 48% of the reactions in the initial GEM. We found Pearson correlation coefficients of 0.35, 0.23 and 0.15 for the meristematic, elongation and maturation zone, respectively, while the Spearman correlation was 0.63, 0.61, and 0.52, respectively. Therefore, the data indicated that the concordance of the transcriptomics with the proteomics data is in line with what has been reported in the literature^33,34^ and highlighted the non-linear relationship between the two (due to the higher values for the Spearman correlation coefficient).

Due to the non-linear relationship between transcript and protein levels, we opted to only compare the structure of the models resulting from the application of RegrEx with transcriptomics and proteomics data. We found that the models for meristematic, elongated, and matured cells from the proteomics data for the scenario Li 1 included 907, 822, and 730 reactions, respectively, in the decoupled case. For the coupled models, these numbers were 632, 832, and 785, respectively. The Jaccard distance to the respective models was the smallest for elongated decoupled models (0.38) and the largest for matured decoupled (0.51). In the case of coupled models, the smallest Jaccard distance was observed for matured cells (0.42), while the largest was for the comparison of meristematic cells (0.49).

### Coupled models facilitated the study of IAA and trans-Zeatin fluxes through the root

In addition, we were interested to inspect how the IAA and trans-Zeatin fluxes were distributed across the different tissues or cell types. Therefore, the multi-tissue models of every tissue or cell type were combined with each other by connecting the respective exchange reactions (i.e., import or export).

Mounting evidence pointed that indole-3-actetate (IAA) is transported through the root towards the tip and back describing a reverse fountain-like shape^1,35,36^. In addition demonstrated in *in-silico* simulations that IAA concentration reaches a local peak at ~0.1 mm away from the root tip corresponding to Birnbaum *et al*.’s definition of developmental stage 1 (0.15 mm away from the root tip)^3,35^. Brunoud *et al*. reported an increase of auxin in the beginning of the elongation zone when investigating the auxin signaling sensor DII-Venus^37^.

Next, we were interested in finding the extent to which the IAA-related observations and simulations could be replicated by the multi-tissue root model. To this end, we formulated a combined biomass reactions that includes the biomass reactions of all coupled models. In the resulting model, we first asked how different are the optimal biomass fluxes (per flux balance analysis) with and without the constraints that the IAA flux is in the reverse fountain-like shape. We found that there was no difference between the two optimal values, indicating that the extracted models supported the known pattern of IAA movement in a reverse fountain-like shape at optimal growth.

**Figure 6:**
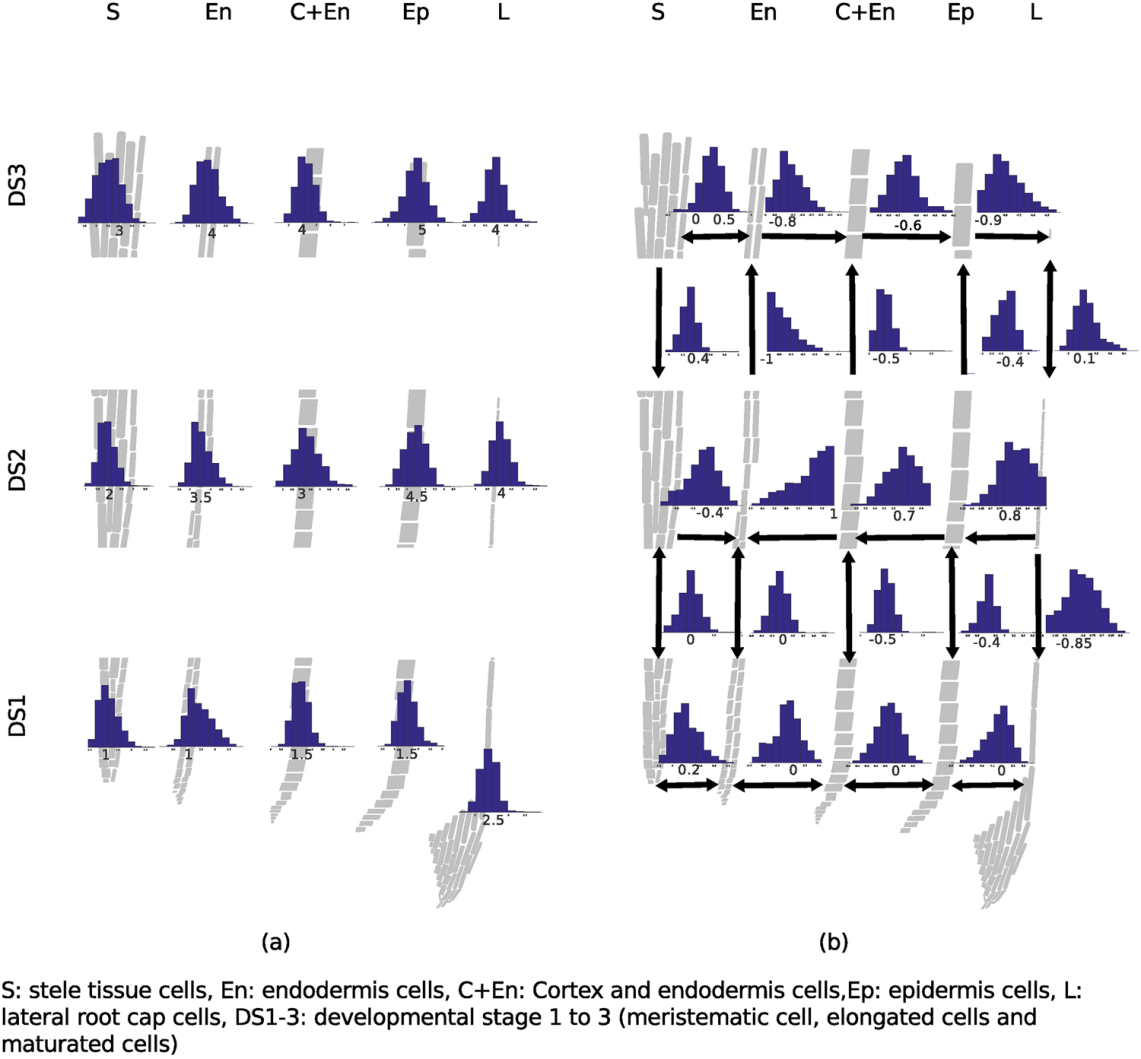
Indole-3-acetate behavior in the root. Fluxes for the extracted models are sampled uniformly by employing the hit-and-run algorithm. (**a**) The flux-sum of IAA in each of the cell types. (**b**) Fluxes through IAA transport reactions. The distribution of samples fluxes determines the preferred direction of the IAA flow when boundaries at optimum steady-state.

To obtain insights about the IAA’s turnover, which can be directly connected to the pool size, we sampled steady-state flux distribution at optimum biomass by using the implementation of Megchelenbrink *et al*.^38^. We then determined the flux-sum as a proxy of IAA’s turnover in each of the cell types^39^. Our findings indicated an increase in the flux-sum at the beginning of the elongation zone compared with the meristematic zone (see Fig. 6a). This corroborated the observations of Brunoud *et al*. about the increase of the IAA concentration in this part of the root^37^. However, the auxin concentration peak close above the cap was not found in our simulations. This could be caused by an averaging effect when considering multiple cell layers at once when modeling developmental stage (due to small resolution).

From Fig. 6b, the steady-state flux distributions revealed that IAA moved predominantly cyclically from one cell type on the periphery to the inner part of the root. For instance, IAA is moving downstream the stele to endodermis cell type of developmental stage 1. It then entered epidermis cells of developmental stage 1, via cortex & endodermis and epidermis cells and finally, it moved back to stele cells of developmental stage 3 via endodermis cell of developmental stage 2.

Similar to the analysis of auxin flux and turnover, we next inspected trans-Zeatin as a representative of the cytokinins (CK). It was detected mainly in the maturated zone of stele, cortex & endodermis, epidermis and lateral root cap cells. Trans-Zeatin was also tracked in the meristematic cortex & endodermis as well as lateral root cap cells (see Supplementary Figure S2). CKs are acting in an antagonistic fashion to auxin and are involved in cell differentiation^28,40^. We wanted to know if and to what extent this behavior was observable in our simulations. Therefore, we analyzed the turnover for trans-Zeatin. Our simulations showed low CK turnover when the turnover of IAA is high: For instance, 0.25 arbitrary unit (a.u.) and 4 a.u. CK’s or IAA’ turnovers in cortex & endodermis or lateral root cap cells of developmental stage 3, respectively, compared with 1.5 a.u. and 1 a.u. of CK’s and IAA’ turnovers in cortex & endodermis or lateral root cap cells of developmental stage 1, respectively (see Supplementary Figure S2a and Fig. 6a). This indicated the antagonistic behavior of CK to IAA, as supported by the negative correlation of −0.3 (p < 0.001) between CK’s and IAA’s turnover from sampled flux distribution of lateral root cap cells of developmental stage 1 or of −0.62 (p < 0.001) of developmental stage 1 (see Supplementary Table S4).

Further experiments are needed to contrast the proposed reverse fountain-like shape of IAA movement in comparison to the predictions of cyclic movement at the majority of optimal steady-state flux distributions as well as for the interplay between auxin and trans-zeatin. Our findings indicate that other constraints, other than optimal growth, are needed to enforce a particular pattern of IAA and CK movement.

## Conclusions

By employing RegrEx^12^ with tissue-specific information given by transcriptomics and metabolomics data, tissue-specific models were extracted from an initial GEM. In parallel, replicates of the initial GEM were interlinked with each other by exchange reactions. From this, RegrEx extracted a multi-tissue model with tissue or cell type specific information provided by the transcriptomics and metabolomics data. This approach took into account that adjacent cell types (of several developmental stages) are autonomous subsystems that communicate with each other, e.g., via exchange of metabolites and other components (e.g. proteins and transcripts).

A comparison of the coupled models revealed that the predicted flux distributions provided at least as good a fit to the transcriptomics data as decoupled tissue-specific models. This was supported by the Pearson as well as the Spearman correlation between fluxes and transcriptomics data (see Supplementary Tables S1, S2 and Fig. 4). Our analysis demonstrated that consideration of multiple cell types may overcome the issue of model overfitting which occurs when single cell types are considered individually. In addition, our modeling strategy resulted in higher model specificity, since the extracted network structures and flux distributions they support differ more for coupled in comparison to decoupled models. Future studies could focus on comparative analysis of other existing approaches for context-specific modeling to consider coupling.

A sampling of flux distributions of the extracted models showed that the imposed coupling leads to a higher specificity of each single (coupled) tissue-specific model, quantified functionally, by a larger discordance between the predicted flux distributions, and structurally, by a larger number of tissue-specific reactions (see Fig. 4). From this fact we concluded that enabling the tissue-specific models to communicate with each other, the resulting multi-tissue model was a more realistic scenario and, thus, more suitable for investigating metabolic processes. The decoupled models in a stand-alone mode needed to exploit further pathways, e.g., because of energy related issues, what was not required any longer when respective metabolic entities were able to be exchanged.

A more application-driven attempt for biological validation was the tracking of IAA and trans-Zeatin (as a representative of cytokinins) fluxes through the root. We showed that our approach allowed to simulate the IAA flux gradient as described in other published results (see Supplementary Table S3). However, we also concluded that other constraints were required to obtain the reverse fountain-like shape for IAA movement, which did not follow from the structure of the model nor from the imposing the optimization of growth. In addition, we showed that our simulation could replicate the antagonistic relationship between IAA and trans-Zeatin observed in experiments.

Altogether, our study revealed that the provided pipeline represented a suitable means to obtain refined tissue-specific models that also consider the proximity of the corresponding tissue without any loss of biological reliability and at higher tissue-specificity of the resulting predictions.

## Methods

### Modeling rationale

Starting from a GEM and a tissue-specific data set, current extraction strategies result in stand-alone tissue-specific models which we refer to as *decoupled*. However, the functionality of tissue-specific metabolic networks may be affected by interactions with others in their spatial proximity. This can be due either to transfer of small molecules (e.g. metabolites), but also by mobility of transcripts and proteins^41^. Therefore, our motivation was to test if the specificity and function-related predictions of tissue-specific models can be improved by extracting interlinked tissue-specific models. Here, we extracted such interlinked tissue-specific models for each developmental stage. We refer to these tissue-specific models as *coupled*, since their extraction considered the exchange of biochemical entities (here, metabolites). The multi-tissue model was then formed by the coupled tissue-specific models. The specificity was then evaluated by comparing the structure of the decoupled with the coupled models, i.e. which reactions were extracted from the initial GEM to form the respective models. Finally, our function-related predictions was based on the comparison of the resulting flux distributions as well as the particular patterns of hormone distribution in the root.

### Genome-scale metabolic model used for tissue-specific model extraction

Here, we used a recently assembled evidence-based GEM of *Arabidopsis thaliana*, called PlantSEED, that covered the entirety of documented metabolic reactions for this model plant^42^. The PlantSEED model included 2841 reactions interconverting 2863 metabolites. For 1523 (i.e., 54%) reactions gene-protein-reaction association rules (GPRs) were available. Due to this high degree of integrity, the PlantSEED model was well-suited for extracting tissue-specific models by incorporating “omics” data sets. Splice variants of genes provided in the model were not considered, since the employed publicly available tissue-specific “omics” data sets did not distinguish between them.

To keep the computational cost low, the blocked reactions were removed from the initial model. To this end, blocked reactions were identified by employing flux variability analysis (FVA) to the GEM at maximum biomass. A reaction that carried a maximum flux smaller than 10^−6^ was considered to be blocked and removed. This reducing step resulted in a model variant with 2199 reactions and 1813 metabolites. The reduced PlantSEED model was then employed to extract metabolic networks specific to stele, endodermis, cortex and epidermis (atrichoblasts) cells as well as to the three developmental stages. In addition, models were extracted that are specific to xylem, phloem and pericycle cells as well as to three developmental stages of the root in its entirety, respectively, without distinguishing between cell types.

### Data used for model extraction and validation

The transcriptomics data used for model extraction were generated with ATH1 GeneChip arrays (Affymetrix, Santa Clara, CA) by Birnbaum *et al*.^3^. The gene expression profiles covered over 22,000 genes and one tissue and four cell types (i.e., stele, endodermis, cortex, epidermis and lateral root cap) of three developmental stages. Since the developmental stages correlated with distance to the tip of the root, Birnbaum *et al*. defined developmental stage 1 as the region between the root tip and 0.15 mm upwards (where the full diameter of the primary root is reached). The developmental stage 2 was given by cells originating from the root section between 0.15 mm to 0.30–0.45 mm away from the root tip. Finally, the section about 0.45 mm to 2 mm far away from the root tip refered to the developmental stage 3. Next to this, more recent transcriptomics data from Li *et al*. were used to extract further models, which were either spatially or temporal resolved^25^. Beyond being more recent, Li *et al*. provided data for stele tissue on a cellular scale, i.e., xylem, phloem and pericylce cell type. In total, there were three data situations resulting in three scenarios, i.e. (a) Birnbaum – spatiotemporal resolved, (b) Li 1 – cell type and (c) Li 2 – developmental stage resolved (see Fig. 1).

In addition, metabolomics data for four metabolites covered by initial model out of 111 annotated metabolites, gathered by Moussaiff *et al*.^26^ were integrated for tissue-specific model refinement, i.e., to enforce that the metabolite is produced or consumed in a given cell type. The metabolomics data were obtained by high-resolution liquid chromatography mass spectroscopy (LC-MS) applied to green fluorescent proteins (GFP) marker lines.

For the biological validation of the resulting tissue-specific models, we employed the proteomics data gathered by Li *et al*. and Petricka *et al*.^10,27^. Following cell-type sorting by GFP markers, the proteomics data were gathered by quantitative mass. The proteomics data sets were either cell type resolved or temporal resolved along the whole root organ. Thus, when validating the extracted models by employing the data sets provided by Li *et al*.^25^, both, fluxes and transcriptomics data of either different cell type or of developmental stages were compared with the corresponding proteomics data sets.

### Overview of RegrEx

The RegrEx^12^ approach was intend to extract context-specific models from a GEM solely by utilizing context-specific data and the ${\ell }_{1}$-norm of the flux distribution. RegrEx has been validated with different data sets and models, and its performance was extensively compared with state-of-the-art approaches for constructing context-specific models^12^, including representatives from the other groups of approaches for context-specific metabolic modeling (e.g. iMAT^18^, FastCORE^43^ and an approach following similar strategy as RegrEx called Lee2012^13^). The performance of RegrEx was evaluated based on the structure of the extracted models, i.e. the number of extracted reactions, the set of shared (core) and exclusive reactions as well as the number of data-orphan reactions (i.e., number of incorporated reactions with non-associated experimental data). RegrEx was also compared to the existing contenders with respect to the similarity to the data used for extracting context-specific models. This comprehensive comparative analyses demonstrated that models extracted by RegrEx were of smallest compactness (i.e. the number of extracted reactions) compared to the models extracted by the other existing approaches. Models extracted by RegrEx also showed the smallest number of core and biggest number of exclusive reactions, demonstrating the context-specificity. RegrEx also resulted in the smallest number of reactions without experimental evidence, i.e. data-orphan reactions compared to the other approaches. In summary, these findings demonstrated that RegrEx is more tissue-specific and of equal or greater compactness compared to the considered approaches.

Next, Robaina-Estévez and Nikoloski were asking for the extent of the discrepancies between predicted flux distribution and the experimental data. Therefore, they considered the Pearson correlation between flux values and the corresponding data for RegrEx as well as for Lee2012^13^ and iMAT^18^. Models extracted by RegrEx showed highest correlation, followed by Lee2012. In a further step, they compared the sets of extracted reactions per context by the Jaccard index. This facilitated the comparison to the findings from FastCORE^43^ on the same data sets and models. As a result, RegrEx was able to capture differences between contexts also with a lower number of extracted reactions compared to the other extracting methods. Altogether, RegrEx was shown to be comparable to “gold-standard” approaches with the benefit that it operated user-independent and in an automated fashion (see further details below). Therefore, RegrEx was the method of choice for our approach.

Before incorporating data into the initial GEM, all transcriptomics data sets were normalized by the maximum value of all considered tissue per gene in accordance to^12^,

1 $${d}_{i,j}=\frac{{t}_{i,j}}{{\rm{\max }}({t}_{i,\forall j})},$$

with *i*, *j* denoting genes and contexts, respectively. By doing this, we ensured that RegrEx does not favor any reactions with higher associated expression values among tissues. The data was then assigned to each reaction in accordance to its corresponding gene-protein-reaction (GPR) association as described in^44^. In a third step, metabolites detected by Moussaiff *et al*.^26^ were enforced to be present in the extracted tissue–specific models. Therefore, for every detected metabolite a respective turnover metabolite was added to the PlantSEED model in accordance with the GIM^3^E approach^16,26^. Furthermore, for each turnover metabolite, we introduced a sink reaction enforced to carry a small non-zero flux of value 10^−07^ to ensure that the respective metabolite and reactions in which it is involved are also included in the (de)coupled context–specific models. In addition, the fluxes through reactions catalyzed by ribulose-1,5-bisphosphate carboxylase/oxygenase (RuBisCo) were forced to be zero, since photosynthesis does not take place in the root.

Subsequently, RegrEx^12^ aimed to find a flux distribution for that the distance to the mapped transcriptomics data is minimized following the idea of the Least Absolute Shrinkage and Selection Operator (LASSO)^45^ approach as it sought to identify a sparse flux distribution compatible with the data (see Equation 2). The resulted flux distribution gave the best concordance to the transcriptomics data. Therefore, reversible reactions were first split into two irreversible reactions. A variable selected one of the two directed reactions. Therefore, RegrEx was formulated as mixed-integer quadratic program (MIQP) in form of,

2 $$\begin{array}{ll}min & \frac{1}{2}\Vert d-{v\Vert }_{2}^{2}+\lambda {\Vert v\Vert }_{1}\\ s.t. & Sv=0\\  & {v}_{i}+{\sigma }_{i}-{x}_{i}{d}_{i}={d}_{i}\\  & {v}_{for,i}+{\sigma }_{for,i}-{x}_{i}{d}_{i}={d}_{i}\\  & {v}_{rev,i}+{\sigma }_{rev,i}-{x}_{i}{d}_{i}={d}_{i}\\  & {v}_{for,i}+{x}_{i}{v}_{max}\le {v}_{max}\\  & {v}_{rev,i}+{x}_{i}{v}_{max}\le 0\\  & {v}_{for,i}+{x}_{i}{v}_{min}\ge {v}_{min}\\  & {v}_{rev,i}+{x}_{i}{v}_{min}\ge 0\\  & {v}_{sink}\ge 1\,\cdot {10}^{-7}\\  & {v}_{min}\le v\le {v}_{max}\\  & {\varepsilon }_{min}\le \varepsilon \le {\varepsilon }_{max}\\  & \varepsilon ,v\in {{\mathbb{R}}}^{n}\\  & x\in {\{0,1\}}^{n}\end{array}$$

with $d,\,v,{\epsilon }$ denoting the transcriptomics data vector (mapped data in accordance to the GPR rules), the flux distribution determined by RegrEx and their discrepancy or error (${\epsilon }=d-v$), respectively. ${v}_{sink}$ gave the sink reactions for every inserted turnover metabolite.

The first line of Equation 2 gave the objective function that was minimized. Its first term ($\frac{1}{2}{\Vert d-v\Vert }_{2}^{2}$) corresponded to the Euclidean distance between the transcriptomics data and the predicted flux values. The second term (*λ*||*v*||_1_ i.e., ${\ell }_{1}$-norm) enforced the actual selection of tissue-specific reactions. The number of extracted reactions can be controlled by the value of the weighting factor $\lambda$. Therefore, from a sequence ranging from 0 to 0.15 with a step size of 0.01, an optimal $\lambda$ was selected with respect to the Pearson correlation between fluxes and the data. The value of $\lambda$ that corresponded to the highest concordance between fluxes and data was considered to be optimal^31^. Therefore, the selected value of $\lambda$ may differ across considered tissues (see Table 5). The flux values of reactions irrelevant for the tissue-specific models were shrunk to zero if they did not contribute to increasing the concordance between transcripts and fitted fluxes. Finally, reactions that carried fluxes of absolute values greater than 10^−6^ are considered to be active and extracted to form the context-specific model. For this reason, the selection of reactions was considered independent of the user and no biological *a priori* knowledge was needed to apply the approach since as no threshold is used on the transcriptomics data.

**Table 5:** Optimal regulation coefficient *λ*.

		decoupled	coupled
xylem		0.15	
phloem		0.15	0.14
pericycle		0.15	
elongation zone		0.12	
maturation zone		0.10	0.12
meristematic zone		0.13	
meristematic zone		0.13	
elongation zone	stele	0.15	0.15
maturation zone		0.13	
meristematic zone		0.13	
elongation zone	endodermis	0.14	0.15
maturation zone		0.15	
meristematic zone		0.07	
elongation zone	cortex &endodermis	0.14	0.15
maturation zone		0.15	
meristematic zone		0.13	
elongation zone	epidermis	0.14	0.15
maturation zone		0.15	
meristematic zone		0.14	
elongation zone	lat root cap	0.14	0.15
maturation zone		0.07	

The underlying principle of RegrEx^12^ is the LASSO approach^45^, in which a regularization term can be controlled by the coefficient $\lambda$. In RegrEx, it also controlled the number of reactions that were extracted from the initial genome-scale model. From a series of $\lambda$ ranging between 0 and 0.15 with a step size of 0.01 an optimal value was given when the Pearson correlation between the predicted flux values and the transcriptomics data was maximum. For all three scenarios Li 1, Li 2 and Birnbaum this was checked for the (de)coupled models.

We had used transcriptomics data to approximate flux phenotype due to the larger coverage in comparison to proteomics data (e.g., the root transcriptomics data in^3,17^ covered 90% of the genome of the *Arabidopsis thaliana* each) compared to proteomics data. There were still an on-going debate about the suitability of transcriptomics data in metabolic modeling (see, for instance, Gygi *et al*. and Moxley *et al*. about the moderate Spearman and Pearson correlations between transcriptomics data and fluxes^33,46^). However, Machado *et al*. showed that predictions supported by transcriptomics data were as good as those based on proteomics data^14^. Since, for the time being this was the only experimentally accessible approach at reasonable costs (e.g., in comparison to labeling experiments and their analysis at genome-scale level), our analysis relied on transcriptomics data.

RegrEx was applied in two subsequent tasks. In a first step, stand-alone decoupled context-specific models were extracted from the initial GEM in accordance to the respective data set (see Fig. 2b). Analogous to this, the initial GEM was replicated and interlinked by adding exchange reactions (see Fig. 2c). To this end, the stoichiometric matrix *S* (Eq. (3)) was built accordingly:

3 $$S=(\begin{array}{cccccc}S1 & \ldots  & \ldots  & \ldots  & \ldots  & \\ \ldots  & S2 & \ldots  & \ldots  & \ldots  & \\ \ldots  & \ldots  & S3 & \ldots  & \ldots  & E\\ \ldots  & \ldots  & \ldots  & \ldots  & \ldots  & \\ \ldots  & \ldots  & \ldots  & \ldots  & Sn & \end{array}).$$

The new stoichiometric matrix comprised all stoichiometric matrices of all context-specific models denoted by *S*_*i*_ with *i = *1, 2, …, *n* (i.e., replicates of the stoichiometric matrix of the initial GEM), as well as the exchange reactions denoted by *E* (see Eq. (4)),

4 $$E=(\begin{array}{ccccccc}-1 & 0 & 0 & \cdots  & 0 & 0 & 0\\ 0 & -1 & 0 & \cdots  & 0 & 0 & 0\\ 0 & 0 & -1 & \cdots  & 0 & 0 & 0\\ \cdots  & \cdots  & \cdots  & \cdots  & \cdots  & \cdots  & \cdots \\ 1 & 0 & 0 & \cdots  & -1 & 0 & 0\\ 0 & 1 & 0 & \cdots  & 0 & -1 & 0\\ 0 & 0 & 1 & \cdots  & 0 & 0 & -1\\ \cdots  & \cdots  & \cdots  & \cdots  & \cdots  & \cdots  & \cdots \\ 0 & 0 & 0 & \cdots  & 1 & 0 & 0\\ 0 & 0 & 0 & \cdots  & 0 & 1 & 0\\ 0 & 0 & 0 & \cdots  & 0 & 0 & 1\end{array}).$$

Exchanged metabolites (denoted by *m*_*j*_^*i*^ where *i* stands for the considered tissue and *j* for the metabolite to be exchanged) were, e.g., water, ammonium, sucrose, cations (i.e., potassium and hydrogen) as well as ions (e.g. chlorid ions)^23^. The considered exchanged metabolites were translocated in accordance to the geometry of the cellular organization. Metabolites only moved from a given context to its adjacent contexts, as sketched in Fig. 2c. From these exchange reactions RegrEx extracted those which are needed to optimize the objective function described above. A biomass reaction was formulated for each (de)coupled context-specific model, an approach already taken in Dal’Molin *et al*.^23^ (see Supplementary Table S5). The resulting model was the multi-context model and its context-specific parts were referring to be coupled (see Fig. 2c).

### Assessing model validity

The model was validated by inspecting the Pearson correlation between the predicted fluxes and the corresponding transcriptomics data. Since transcript data may not be good proxies for fluxes as mentioned above, we employed another strategy for validation; namely, we randomly generate flux distributions for the extracted tissue-specific models. To this end, the Artificial Centered Hit-and-Run (ACHR) sampling method was employed^47^ with the implementation provided by^31^ and^38^. In Figure S1 the validation strategy was illustrated: for two tissue-specific models found by RegrEx, named model 1 and 2, 2,500 flux distributions were uniformly sampled. Subsequently, the Pearson correlation coefficient was calculated for every pair of reactions included in the considered model over the sampled flux distributions. The resulting correlation matrices from the two models were, in turn, compared by determining the *R*_*v*_-coefficient^48^. The *R*_*v*_-coefficient took values between zero and one; similarly to the Pearson correlation coefficient, a value for the *R*_*v*_-coefficient that was close to one implied a stronger correspondence between the compared correlation matrices or metabolic models. Finally, we also provided a correlation-based comparison of the proteomics data to the randomly sampled feasible flux distributions. The distributions of flux sum for IAA as well as trans-Zeatin were determined as the sum of fluxes multiplied by the respective stoichiometry around a metabolic pool for each of the sampled flux distributions.

All scripts were implemented in MATLAB scripting language (MATLAB Release 2015a, The MathWorks, Inc., Natick, Massachusetts, United States^49^) under usage of the COBRA toolbox 2^50^ and the cplex-solver^51^ provided by the TOMLAB Optimization Environment by Tomlab Optimization^52^. All implementations used in this study are available from the corresponding author upon request.

## Electronic supplementary material

Supplementary Material
