# Supplementary material for: Integration of large-scale data for extraction of integrated Arabidopsis root cell-type specific models
Source: Sci Rep. 2018 May 21;8:7919. doi: 10.1038/s41598-018-26232-8 (PMC5962614; doi:10.1038/s41598-018-26232-8)
Supplement: Supplementary file 1 — Supplementary Material [file 41598_2018_26232_MOESM1_ESM.pdf]

Supplementary File:

Integration of large-scale data for extraction of integrated *Arabidopsis* root cell-type specific models

Michael Scheunemann[1,3], Siobhan M. Brady[2], Zoran Nikoloski[1,3,*]

[1]*Systems Biology and Mathematical Modeling, Max Planck Institute of Molecular Plant Physiology, Potsdam-Golm, Germany*
[2]*Department of Plant Biology and Genome Center, University of California, Davis, CA 95616 USA*
[3]*Bioinformatics Group, Institute of Biochemistry and Biology, University of Potsdam, Potsdam-Golm, Germany*

[*] *Correspondence should be addressed to Z.N. (email: nikoloski@mpimp-golm.mpg.de)*

Supplementary Figures

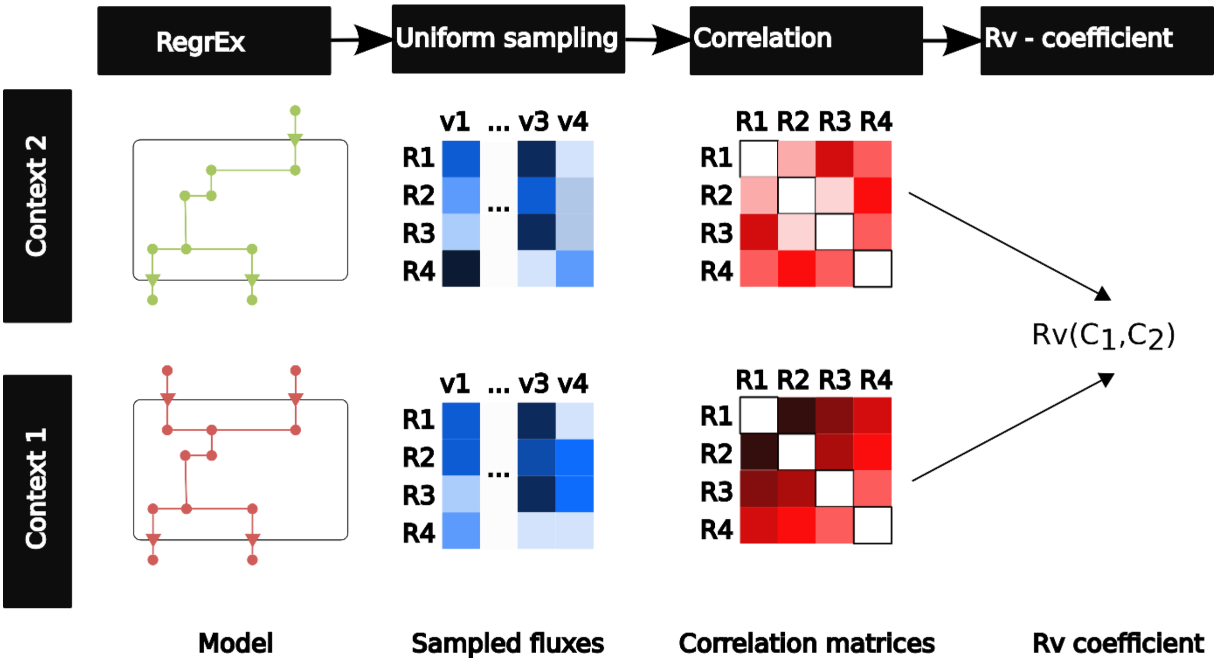

Figure S1: Sampling pipeline. For functional biological validation flux values for all extracted models were uniformly sampled. The sampled flux distributions were then pairwise compared by employing the Pearson correlation coefficient. Finally, for both, decoupled and coupled models the correlation matrices were compared with each other by determining the R_v coefficient.

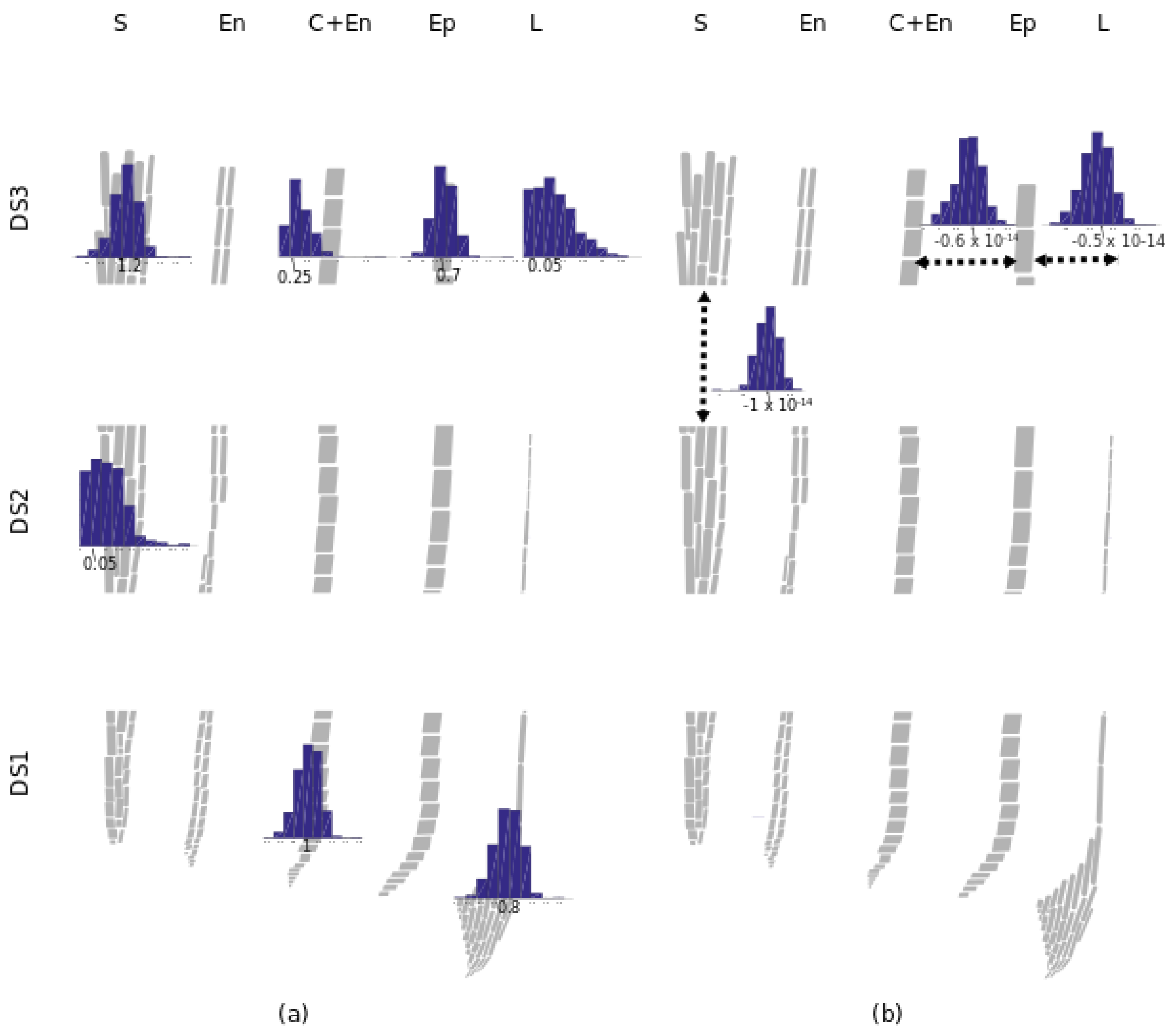

S: stele tissue cells, En: endodermis cells, C+En: Cortex and endodermis cells,Ep: epidermis cells, L: lateral root cap cells, DS1-3: developmental stage 1 to 3 (meristematic cell, elongated cells and maturated cells)

Figure S2: Cytokinin behavior in the root model. Fluxes from the extracted models were uniform sampled by employing the hit-and-run algorithm at optimal biomass. (a) The flux-sum of cytokinin in each cell-type were it was detected. (b) Fluxes through cytokinin transport reaction.

Supplementary Tables

Table S1: Concordance between flux distributions and the transcriptomics data using Spearman correlation coefficient. For all three scenarios Li 2, Li 1 and Birnbaum the flux distributions are compared with the respective transcriptomics data by determining the Spearman correlation coefficient. All presented correlations were significant at level 0.001 except those for that the p-value is given in brackets.

		decoupled	coupled
elongation zone		0.38	0.33
maturation zone		0.35	0.29
meristematic zone		0.07 (0.170)	0.00 (1.000)
xylem		0.37	0.37
phloem		0.35	0.32
pericycle		0.36	0.39
elongation zone		0.24	0.24
maturation zone	stele	0.33	0.32
meristematic zone		0.46	0.49
elongation zone		0.14 (0.003)	0.15 (0.001)
maturation zone	endodermis	0.40	0.42
meristematic zone		0.42	0.43
elongation zone	cortex	0.13 (0.004)	0.22
maturation zone	+	0.31	0.33
meristematic zone	endodermis	0.39	0.38
elongation zone		0.12 (0.011)	0.06 (0.223)
maturation zone	epdermis	0.29	0.30
meristematic zone		0.39	0.37
elongation zone		0.32	0.32
maturation zone	lat root cap	0.35	0.38
meristematic zone		0.49	0.53

Table S2: Structural and functional comparison of de/coupled context-specifc models. For three scenarios the decoupled models were compared with coupled models: scenario Birnbaum (only transcriptomics data from Birnbaum *et al.*[1] are used), scenario Li 1 in which transcriptomics data for different cell types from Li *et al.*[2] are considered and scenario Li 2 in which the gene expression data for three developmental stages were considered. For the comparison the Pearson as well as the Spearman correlation was used. This table belongs to Figure 4. Bold font indicates values corresponding to the decoupled models, whereas the italic font corresponds to the coupled models.

(a) Scenario Li 1

Jaccard distances

	xylem	phloem	pericycle
xylem		*0.35*	*0.42*
phloem	**0.30**		*0.34*
pericycle	**0.31**	**0.27**	

Correlation flux values (Pearson)

	xylem	phloem	pericycle
xylem		*0.65*	*0.61*
phloem	**0.72**		*0.76*
pericycle	**0.74**	**0.79**	

Correlation between flux values (Spearman)

	xylem	phloem	pericycle
xylem		*0.67*	*0.63*
phloem	**0.66**		*0.69*
pericycle	**0.72**	**0.69**	

(b) Scenario Li2

Jaccard distances

	elong	mat	mer
elong		*0.47*	*0.51*
mat	**0.37**		*0.60*
mer	**0.28**	**0.34**	

Correlation between flux values (Pearson)

	elong	mat	mer
elong		*0.46*	*0.40*
mat	**0.61**		*0.25*
mer	**0.56**	**0.55**	

Correlation between flux values (Spearman)

	elong	mat	mer
elong		*0.55*	*0.46*
mat	**0.60**		*0.36*
mer	**0.63**	**0.62**	

(c) Scenario Birnbaum

Jaccard distances

	meristematic	elongated	maturated	
meristematic		*0.33*	*0.39*	stele
elongated	**0.18**		*0.35*	
maturated	**0.27**	**0.20**		
meristematic		*0.39*	*0.44*	endodermis
elongated	**0.20**		*0.31*	
maturated	**0.29**	**0.26**		
meristematic		*0.30*	*0.35*	cortex + endodermis
elongated	**0.29**		*0.35*	
maturated	**0.34**	**0.25**		
meristematic		*0.34*	*0.38*	epidermis
elongated	**0.28**		*0.40*	
maturated	**0.26**	**0.17**		
meristematic		*0.32*	*0.42*	lat root cap
elongated	**0.29**		*0.38*	
maturated	**0.25**	**0.29**		

Correlation between flux values (Pearson)

	meristematic	elongated	maturated	
meristematic		*0.72*	*0.69*	stele
elongated	**0.80**		*0.70*	
maturated	**0.80**	**0.87**		
meristematic		*0.56*	*0.48*	endodermis
elongated	**0.58**		*0.54*	
maturated	**0.66**	**0.60**		
meristematic		*0.65*	*0.65*	cortex + endodermis
elongated	**0.65**		*0.67*	
maturated	**0.63**	**0.69**		
meristematic		*0.75*	*0.66*	epidermis
elongated	**0.89**		*0.79*	

maturated	**0.83**	**0.85**		
meristematic		*0.76*	*0.70*	lat root cap
elongated	**0.76**		*0.77*	
maturated	**0.72**	**0.78**		

Correlation between flux values (Spearman)

	meristematic	elongated	maturated	
meristematic		*0.64*	*0.63*	stele
elongated	**0.74**		*0.61*	
maturated	**0.71**	**0.82**		
meristematic		*0.57*	*0.46*	endodermis
elongated	**0.67**		*0.59*	
maturated	**0.65**	**0.65**		
meristematic		*0.69*	*0.63*	cortex + endodermis
elongated	**0.68**		*0.65*	
maturated	**0.66**	**0.71**		
meristematic		*0.67*	*0.63*	epidermis
elongated	**0.79**		*0.65*	
maturated	**0.76**	**0.84**		
meristematic		*0.74*	*0.63*	lat root cap
elongated	**0.72**		*0.66*	
maturated	**0.71**	**0.72**		

Table S3: FVA of IAA transport reactions. For the IAA transport reactions a flux variability analysis (FVA) was performed. The minimum flux through reaction is given by minFlux and the maximum flux is denoted by maxFlux.

reaction name	minFlux	maxFlux
cpd00703_c0_inter_cellstage_1_STELE	-1	0.99
cpd00703_c0_inter_cellstage_2_STELE	-1	0.99
cpd00703_c0_inter_celltype_stage_steleendo_1_STELE	-1	0.99
cpd00703_c0_inter_celltype_stage_steleendo_2_STELE	-1	1
cpd00703_c0_inter_celltype_stage_steleendo_3_STELE	-0.99	1
cpd00703_c0_inter_cellstage_1_ENDO	-1	1
cpd00703_c0_inter_cellstage_2_ENDO	-1	1
cpd00703_c0_inter_celltype_stage_endostele_1_ENDO	-0.99	1
cpd00703_c0_inter_celltype_stage_endocortexendo_1_ENDO	-1	1
cpd00703_c0_inter_celltype_stage_endostele_2_ENDO	-1	1
cpd00703_c0_inter_celltype_stage_endocortexendo_2_ENDO	-1	1
cpd00703_c0_inter_celltype_stage_endostele_3_ENDO	-1	0.99
cpd00703_c0_inter_celltype_stage_endocortexendo_3_ENDO	-1	1
cpd00703_c0_inter_cellstage_1_CORTEX	-1	1
cpd00703_c0_inter_cellstage_2_CORTEX	-1	1
cpd00703_c0_inter_celltype_stage_cortexendoendo_1_COR	-1	1
cpd00703_c0_inter_celltype_stage_cortexendoepidermis_1_COR	-1	1
cpd00703_c0_inter_celltype_stage_cortexendoendo_2_COR	-1	1
cpd00703_c0_inter_celltype_stage_cortexendoepidermis_2_COR	-1	1
cpd00703_c0_inter_celltype_stage_cortexendoendo_3_COR	-1	1
cpd00703_c0_inter_celltype_stage_cortexendoepidermis_3_COR	-1	1
cpd00703_c0_inter_cellstage_1_EPI	-1	1
cpd00703_c0_inter_cellstage_2_EPI	-1	1
cpd00703_c0_inter_celltype_stage_epidermiscortexendo_1_EPI	-1	1
cpd00703_c0_inter_celltype_stage_epidermislatrootcap_1_EPI	-0.99	1
cpd00703_c0_inter_celltype_stage_epidermiscortexendo_2_EPI	-1	1
cpd00703_c0_inter_celltype_stage_epidermislatrootcap_2_EPI	-1	1
cpd00703_c0_inter_celltype_stage_epidermiscortexendo_3_EPI	-1	1
cpd00703_c0_inter_celltype_stage_epidermislatrootcap_3_EPI	-1	1
cpd00703_c0_inter_cellstage_1_LRC	-1	0.99
cpd00703_c0_inter_cellstage_2_LRC	-1	1
cpd00703_c0_inter_celltype_stage_latrootcapepidermis_1_LRC	-1	0.99
cpd00703_c0_inter_celltype_stage_latrootcapepidermis_2_LRC	-1	1
cpd00703_c0_inter_celltype_stage_latrootcapepidermis_3_LRC	-1	1

Table S4: Turnovers of auxin and cytokinin. From the sampled flux distribution the respective flux sum is calculated as a proxy for auxin (IAA) and cytokinin (CK). The IAA's and Ck's turnover are then compared with each for cortex & endo cells (cortexendo) and lateral root cap cells (lrc) by employing the Pearson and Spearman correlation. Bold font indicates denotes the Pearson correlation, whereas the italic font corresponds to the Spearman correlation. All correlations are significant at a level of p = 0.001.

		IAA		CK	
		cortexendo (stage 1)	cortexendo (stage 3)	cortexendo (stage 1)	cortexendo (stage1)
IAA	cortexendo (stage 1)			*-0.25*	
	cortexendo (stage 3)				*-0.43*
CK	cortexendo (stage 1)	**-0.48**			
	cortexendo (stage 3)		**-0.30**		
		lrc (stage 1)	lrc (stage 3)	lrc (stage 1)	lrc (stage 3)
IAA	lrc (stage 1)			*-0.60*	
	lrc (stage 3)				*-0.16*
CK	lrc (stage 1)	**-0.62**			
	lrc (stage 3)		**-0.23**		

Table S5: Biomass composition. Metabolites required to produce biomass, such as amino acids (precursor for proteins), as well as the respective stoichiometric coefficients that are used to in the analyses.

Metabolite	Stoichiometry
Orthophosphate	30,000
H2O	-30,000
H	30,000
ATP	-30,022
ADP	30,000
L-Glutamine	-0,081
L-Glutamate	-0,093
UTP	-0,020
L-Methionine	0,000
L-Alanine	-0,025
GTP	-0,023
L-Aspartate	-0,038
L-Lysine	-0,016
L-Asparagine	-0,091
Glycine	-0,032
L-Arginine	-0,039
CTP	-0,020
L-Serine	-0,050
L-Tryptophan	-0,008
L-Phenylalanine	-0,019
L-Tyrosine	-0,008
L-Threonine	-0,020
Sucrose	-0,031

References

1 Birnbaum, K. *et al.* A gene expression map of the Arabidopsis root. *Science* **302**, 1956-1960, doi:10.1126/science.1090022 (2003).

2 Li, S., Yamada, M., Hang, X. W., Ohler, U. & Benfey, P. N. High-Resolution Expression Map of the Arabidopsis Root Reveals Alternative Splicing and lincRNA Regulation. *Dev Cell* **39**, 508-522, doi:10.1016/j.devce1.2016.10.012 (2016).
